# Protection of Mice against Lethal Challenge with 2009 H1N1 Influenza A Virus by 1918-Like and Classical Swine H1N1 Based Vaccines

**DOI:** 10.1371/journal.ppat.1000745

**Published:** 2010-01-29

**Authors:** Balaji Manicassamy, Rafael A. Medina, Rong Hai, Tshidi Tsibane, Silke Stertz, Estanislao Nistal-Villán, Peter Palese, Christopher F. Basler, Adolfo García-Sastre

**Affiliations:** 1 Department of Microbiology, Mount Sinai School of Medicine, New York, New York, United States of America; 2 Global Health and Emerging Pathogens Institute, Mount Sinai School of Medicine, New York, New York, United States of America; 3 Department of Medicine, Mount Sinai School of Medicine, New York, New York, United States of America; Erasmus Medical Center, Netherlands

## Abstract

The recent 2009 pandemic H1N1 virus infection in humans has resulted in nearly 5,000 deaths worldwide. Early epidemiological findings indicated a low level of infection in the older population (>65 years) with the pandemic virus, and a greater susceptibility in people younger than 35 years of age, a phenomenon correlated with the presence of cross-reactive immunity in the older population. It is unclear what virus(es) might be responsible for this apparent cross-protection against the 2009 pandemic H1N1 virus. We describe a mouse lethal challenge model for the 2009 pandemic H1N1 strain, used together with a panel of inactivated H1N1 virus vaccines and hemagglutinin (HA) monoclonal antibodies to dissect the possible humoral antigenic determinants of pre-existing immunity against this virus in the human population. By hemagglutinination inhibition (HI) assays and vaccination/challenge studies, we demonstrate that the 2009 pandemic H1N1 virus is antigenically similar to human H1N1 viruses that circulated from 1918–1943 and to classical swine H1N1 viruses. Antibodies elicited against 1918-like or classical swine H1N1 vaccines completely protect C57B/6 mice from lethal challenge with the influenza A/Netherlands/602/2009 virus isolate. In contrast, contemporary H1N1 vaccines afforded only partial protection. Passive immunization with cross-reactive monoclonal antibodies (mAbs) raised against either 1918 or A/California/04/2009 HA proteins offered full protection from death. Analysis of mAb antibody escape mutants, generated by selection of 2009 H1N1 virus with these mAbs, indicate that antigenic site Sa is one of the conserved cross-protective epitopes. Our findings in mice agree with serological data showing high prevalence of 2009 H1N1 cross-reactive antibodies only in the older population, indicating that prior infection with 1918-like viruses or vaccination against the 1976 swine H1N1 virus in the USA are likely to provide protection against the 2009 pandemic H1N1 virus. This data provides a mechanistic basis for the protection seen in the older population, and emphasizes a rationale for including vaccination of the younger, naïve population. Our results also support the notion that pigs can act as an animal reservoir where influenza virus HAs become antigenically frozen for long periods of time, facilitating the generation of human pandemic viruses.

## Author Summary

Influenza A viruses generally infect individuals of all ages and cause severe respiratory disease in very young children and elderly people (>65 years). However, the 2009 pandemic H1N1 virus infection is predominantly seen in children and adults (<35 years of age), but rarely in people older than 65 years of age. Recent serological studies indicate that older people carry antibodies that recognize the 2009 H1N1 virus. This suggests that they may have been exposed to or vaccinated with an influenza virus similar to 2009 H1N1 virus. In this study, we wanted to identify the older H1N1 virus(es) that may confer protection to the elderly population. Using 11 different inactivated influenza A viruses that have circulated between 1918 to 2007, we immunized mice and challenged them with a lethal dose of the 2009 novel H1N1 virus. We find that mice vaccinated with human H1N1 viruses that circulated in 1918 and in 1943 were protected from the 2009 H1N1 virus. Also, the 1976 swine origin H1N1 virus, against which nearly 40 million people were immunized in 1976 in the United States, protects mice from death by the 2009 H1N1 virus. This indicates that people carrying antibodies against H1N1 viruses that circulated between 1918–1943 and to the 1976 swine origin H1N1 virus are likely to be protected against the 2009 pandemic H1N1. Importantly, our data underscores the significance of vaccinating people under 35 year of age, since the majority of them do not have protective antibodies against the 2009 H1N1, and provide a possible mechanism by which pandemic viruses could arise from antigenically frozen influenza viruses harbored in the swine population.

## Introduction

Influenza A viruses (IAV), members of the *Orthomyxoviridae* family, cause severe respiratory diseases in humans with an average mortality rate of 36,000/year in the United States alone [1]. Apart from yearly seasonal outbreaks, IAV can cause frequent epidemics and occasional pandemics in humans [2],[3]. Vaccination has been one of the most effective means of protection against IAV. Vaccine induced production of antibodies against the viral surface glycoprotein hemagglutinin (HA) is crucial for immune protection [4]. The HA plays a critical role in the virus life cycle by mediating virus binding to sialic acid containing receptors on the cell surface and fusion of viral and endosomal membranes, leading to viral entry into the host cell [2],[5]. HA-specific antibodies have been demonstrated to block the IAV infection by preventing receptor binding and/or fusion. However, the HA protein, due to antibody mediated immune selection pressure, undergoes rapid antigenic evolution by accumulation of mutations (“antigenic drift”) and through genetic reassortments of segments (“antigenic shift”). In the 20^th^ century, influenza virus caused three pandemics in humans: 1918 “Spanish influenza” (H1N1), 1957 “Asian influenza” (H2N2) and 1968 “Hong Kong influenza” (H3N2) [6],[7]. In April 2009 the Centers for Disease Control and Prevention (CDC) of the United States of America announced the detection of a novel strain of influenza virus in humans. Further investigation revealed that this novel virus derived its genes from viruses circulating in the pig population. Due to sustained human-to-human transmission of this novel virus throughout the world on June 11th, the World Health Organization (WHO) raised the worldwide pandemic alert level to phase 6 (e.g. ongoing global spread and community level outbreaks in multiple parts of world). All of the past pandemics and the recent 2009 swine-origin IAV H1N1 pandemic have been caused by IAV strains carrying an antigenically novel HA segment in populations immunologically naïve to that particular HA.

Apart from humans, IAV can infect a variety of species including poultry, aquatic birds, horses, pigs, dogs and seals [8],[9]. Aquatic wild birds are considered to be natural reservoirs of different subtypes of IAV, and pigs, in addition to harbor swine influenza virus strains, are thought to serve as “mixing vessels” for genetic reassortments of viral segments between avian and human influenza virus strains [8],[10]. Sometime during the 1918–20 pandemic, IAV H1N1 resembling the 1918 pandemic virus was introduced into the domestic swine population [11],[12]. Until today, these H1N1 viruses, referred as classical swine H1N1, have circulated in the swine population with relatively modest changes in HA antigenicity [13]. In contrast to HA antigenic stasis in swine, descendants of the 1918 virus circulated in humans with considerable antigenic drift in HA, until they were replaced by the 1957 H2N2 pandemic virus [4]. In 1978, an H1N1 virus resembling a late 1950's human H1N1 virus reemerged in the human population [14],[15]. From 1977 to the present day, both H3N2 and H1N1 viruses co-circulate in humans. In addition, in 1976, a swine H1N1 IAV (classical H1N1) outbreak was reported among soldiers at the Fort Dix Army base in New Jersey [16],[17]. Because no H1N1 viruses had circulated in humans since 1957, the Fort Dix outbreak raised fears of an H1N1 pandemic. Therefore, a vaccine based upon an inactivated A/NJ/76 (H1N1) virus was developed and administered to nearly 40 million people in the United States. However, the 1976 swine H1N1 virus did not cause a pandemic and no infections were reported outside of Fort Dix.

Although several infections with swine IAV have been reported in humans, such infections have been rare [18],[19],[20],[21]. Surprisingly, the current 2009 pandemic H1N1 has proved to be very efficient in human-to-human transmission compared to previous swine influenza viruses. Apart from lack of pre-existing immunity against this virus in most humans, it is still unclear what viral genetic factors contribute to this higher transmission rate. It is interesting to note that recent epidemiological data indicate a higher rate of confirmed 2009 H1N1 infection in individuals younger than 18 years of age as compared to older individuals [22]. It is speculated that some of the older population may have been exposed to a virus antigenically similar to 2009 H1N1. Nevertheless, it is currently unknown which specific virus or viruses that circulated in the past and during what years, might be responsible for this apparent serum cross-reactivity reported in the older population (above 65 years of age) [23],[24],[25].

In this study, using a lethal mouse model system of the current 2009 H1N1 virus, we tested the efficacy of a panel of 11 different virus vaccines spanning from 1918 to 2009. We demonstrate that mice immunized with inactivated vaccines based on human H1N1 virus from 1918 to 1943 or based on classical swine H1N1 viruses confer complete protection from death against a lethal challenge with the 2009 H1N1 virus. In contrast, vaccination of mice with inactivated human H1N1 viruses isolated after 1950, including contemporary H1N1 viruses, offered only partial protection and resulted in greater morbidity due to 2009 H1N1 virus infection. In agreement with vaccination/challenge studies, pre-challenge sera from mice vaccinated with 1918, and classical swine antigens show neutralization of hemagglutination of 2009 H1N1 virus. In addition, we isolated anti-HA specific monoclonal antibodies (mAb) that have cross-reactivity against 1918 and 2009 H1N1 HA's. In challenge experiments, these mAbs offer complete protection against death by the 2009 H1N1 virus following passive immunization. Analysis of escape mutant viruses selected in the presence of these mAbs mapped to antigenic site Sa. Together, these data suggest that the novel 2009 pandemic H1N1 virus shares a great level of antigenic similarity to the 1918 virus and that site Sa is highly conserved in these two viruses. These results agree well with epidemiological data which indicate that the older population (age >65) are less susceptible to the 2009 H1N1 [24],[25],[26] and with data arising from two recent studies that demonstrated high prevalence of cross-neutralizing antibodies in people born before 1940 [27],[28]. Taken together, our observations provide a rationale for the protection observed in the older population to the 2009 H1N1 pandemic virus and the greater susceptibility seen in younger individuals. Thus, our data indicate that individuals that have been previously exposed to and contain antibodies against 1918-like H1N1 viruses or classical swine H1N1 are likely to be protected against the novel swine-origin 2009 H1N1 virus.

## Materials and Methods

### Ethics statement

All animal procedures performed in this study are in accordance with Institutional Animal Care and Use Committee (IACUC) guidelines, and have been approved by the IACUC of Mount Sinai School of Medicine.

### Cell lines

Human embryonic kidney (293T) cells were maintained in DMEM supplemented with 10% FBS and 1000u/ml penicillin/streptomycin. Madin–Darby canine kidney (MDCK) cells were maintained in MEM supplemented with 10% FBS and penicillin/streptomycin. Reagents for cell culture were purchased from Gibco Life Technologies.

### Virus strains

H1N1 viruses used in this study are as follows: A/Swine/Iowa/30 (Sw/30), A/Puerto Rico/8/34 -MSSM (PR8), A/Weiss/43 (Wei/43), A/New Jersey/8/1976 (NJ/76), A/USSR/92/77 (USSR/77), A/Houston/20593/84 (Hou/84), A/Texas/36/1991 (Tx/91), A/Brisbane/59/2007 (Bris/59/07), A/California/04/2009 (Cal/09) and A/Netherlands/602/2009 (Neth/09). A/Northern Territory/60/1968 (NT/68) and A/Brisbane/10/2007 (Bris/10/07) were used as H3N2 controls. All experiments involving 2009 H1N1 viruses were conducted under Biosafety level 3 (BSL3) conditions for animal work and Biosafety level 2 with BSL3 practices laboratory conditions for *in vitro* work, in accordance with guidelines of the Centers for Disease Control and Prevention. Cal/09 and Neth/09 virus stocks used for mice experiments were all grown in MDCK cells. All other viruses were grown in 10d old eggs at 37°C for 2–3 days.

### Rescue of Cal/09 vaccine strain

The recombinant influenza A virus carrying HA/NA from Cal/09 and the internal six genes from PR8, referred to as Cal/09 6:2 in the reminder of the study, was generated as described previously [29]. Briefly, 293T cells were transfected with eight ambisene pDZ vectors encoding the 8 viral genes and proteins and 24 h post-transfection, the supernatant was inoculated into 8-day-old embryonated chicken eggs. The allantoic fluid was harvested after 3 days of incubation at 35°C. The rescued virus was plaque purified in MDCK cells and re-grown in 10-day-old embryonated chicken eggs.

### Vaccine preparation

All viruses used to prepare inactivated vaccines were grown in 10d old eggs at 37°C for 2 days. After the clarification of debris in the allantoic fluid by low speed centrifugation, the viruses were pelleted on a 30% sucrose cushion by centrifugation at 25,000 rpm for 2hr. The viral pellet was resuspended in calcium borate buffer pH = 7.0 (143mM Sodium chloride, 10mM Calcium chloride, 20mM Boric acid 2.5mM sodium borate) at 1mg/ml concentration calculated by the Bradford method (Bio-Rad laboratories, Hercules, CA) and the virus was inactivated by formaldehyde (0.9% final conc.). For the Cal/09 vaccination group, in place of wild type virus, a Cal/09 6:2 was used (described above). For the 1918 virus-based vaccine, virus-like particles (VLP) were used for vaccination. VLPs were produced by co-transfecting HA (A/South Carolina/1/18) and NA (A/Brevig Mission/1/18) into 293T cells as previously described [30]. Released VLPs were purified on a 30% sucrose cushion. Vaccine doses used were based on the amount of total protein concentration measured by the Bradford method.

### Generation of HA-specific mouse monoclonal antibodies

Pandemic H1N1 2009 and 1918 HA-specific mouse mAbs were generated by the hybridoma shared research facility at Mount Sinai School of Medicine, New York, NY. For the generation of 2009 H1N1 HA-specific antibodies, 6-week-old BALB/C mice were infected intranasally with 5×10^4^ pfu of influenza virus Cal/09. Four weeks later, the mice were given 2×10^7^ pfu of Cal/09 6:2 intravenously to boost Cal/09 HA-specific B cells. Three days after the boost the spleen was harvested and B cells from the spleen were fused with SP2/0 myeloma cells by the addition of polyethylene glycol. The supernatants of the resulting hybridomas were tested by immunofluorescence staining on 293T cells transfected with pDZ-HA (Cal/09). Positive hybridomas were subcloned and re-tested. To generate 1918 virus HA-specific antibodies, mice were immunized by DNA vaccination with pCAGGS-HA of the influenza A/South Carolina/1/18 H1N1 virus and then boosted with whole inactivated virus as above. Hemagglutination inhibition (HI) assays were used to select cross-reactive antibodies against homologous HA using 1918 VLPs. The 1918 HA-specific mAbs 6B9 and 39E4 correspond to isotype IgG2a and the Cal04/09 mAb 29E3 correspond to an IgG2b isotype. The mAbs were isotyped using the IsoStrip kit (Roche, Indianapolis, IN) and purified using a Protein A sepharose column.

### Hemagglutination inhibition assay

Mouse sera were inactivated using a trypsin-heat-periodate treatment as previously described [31]. Briefly, one volume of sera was mixed with half a volume of trypsin 8 mg/ml (Sigma-Aldrich, St. Louis, MO) in 0.1 M phosphate buffer, pH 8.2 and then incubated for 30 min at 56°C. The samples were allowed to cool to room temperature (RT) and were mixed with 3 volumes of 0.11 M metapotassium periodate and incubated at RT for 15 min. Three volumes of 1% glycerol saline were then added and mixed with the samples and further incubated for 15 min at RT. The samples were diluted to a final 1∶10 dilution by adding and mixing 2.5 volumes of 85% saline. HI assays of mAbs and sera were conducted following standard protocols [30]. Two-fold serial dilutions of sera or mAbs were mixed and pre-incubated in 96-well plates for 30 min at 4°C with 8 HA units of virus per well. Turkey red blood cells were added to a final concentration of 0.25%, and the plate was incubated on ice for 30 min. Hemagglutination inhibition (HI) titers of sera were determined as the highest dilution that displayed hemagglutinating activity. Specific HI activity of mAbs was calculated as the lowest concentration of mAb that displayed hemagglutinating activity.

### Generation of mAb escape mutants

mAb escape mutants were generated as previously described [32]. Briefly, Cal/09 (6:2) virus was incubated with excess monoclonal antibody for 1hr at room temperature. The virus-mAb mix was inoculated into 10 day old embryonated eggs and incubated at 37°C for 48 hrs. The virus (allantoic fluid) grown under this conditions was harvested and re-tested for loss of HI activity against the same mAb used for selection. Mutations in the HA responsible for the Ab escape were identified by direct sequencing of vRNA obtained by RT-PCR.

### Mice experiments

#### Infections, body weight loss and survival

BALB/C or C57B/6 mice (Jackson Laboratories, Bar Harbor, ME) were anesthetized with ketamine-xylaxine and intranasally infected with the indicated dose of virus diluted in 50 µl of PBS. Body weight and survival were measured every day for 14 days. All mouse experiments were carried in strict accordance with institutional protocols. Mice showing more than 25% of body weight loss were considered to have reached the experimental end point and were humanely euthanized.

#### Lung titers

Lungs of infected mice were excised on indicated days post-infection and homogenized using a mechanical homogenizer (MP Biochemicals, Solon, OH). The viral titers in the homogenates were quantified by plaque assay on MDCK cells. Each data point presents the average titer from 2–3 mice.

#### Determination of LD_50_ for Neth/09

Female C57B/6 mice (9 weeks old) were anesthetized with ketamine-xylaxine and intranasally infected with doses 5×10^2^, 5×10^3^, 5×10^4^, 5×10^5^ or 5×10^6^ pfu virus in 50 µl (n = 5/group). The mice were monitored daily for survival and body weight loss over a period of 14 day post infection (p.i.) Mice showing more than 25% of body weight loss were considered to have reached the experimental end point and were euthanized. LD_50_ were calculated by the Reed & Muench method [33].

#### Vaccination and challenge

Five-week-old female C57B/6 were immunized with 15 µg of inactivated virus or 1918 VLP by intramuscular injection in the hind leg. Two-weeks later the mice were boosted with the same amount of inactivated virus. Four weeks after the initial immunization mice were challenged with Neth/09 at a dose equivalent to 50 LD_50_ (7.9×10^5^ pfu). Survival and body weight loss were monitored for 14 days. Mice were 9–10 week old at the time of lethal challenge.

#### Passive immunization and challenge

Monoclonal antibodies 6B9 and 39E4 were generated against influenza A/South Carolina/1/18 (H1N1) virus (1918) HA, and monoclonal antibody 29E3 was raised against Cal/09 HA. Nine-week-old C57B/6 mice were passively immunized with a total 150 µg of indicated monoclonal antibody (intraperitoneally) or 200 µl of polyclonal sera from mice previously infected with Cal/09, 24h prior to lethal challenge. A mAb (isotype IgG2a) raised against an unrelated viral protein, Nipah W, was administered as control. The mice were challenged with Neth/09 at a dose equivalent to 50 LD_50_. Survival and body weight loss were monitored for 14 days post challenge (p.c.), and lung viral titers were obtained at days 3 to 6 p.c.

#### Control group

All of the vaccinated and passively immunized mice were challenged at the same time. A single group of control mice were used for comparison in both experiments and refer to either “No vaccination” or “No Ab” controls shown below.

### Homology modeling

The structural models for HA of Cal//09 and Bris/59/07 were generated using structural automated protein structure homology-modeling prediction server (Swiss-Model) with best fitting templates (PDB: 1ruy for Cal/09 and 1rvx for Bris/59/07) [34]. The structure of 1918 HA was obtained from PDB (PDB ID: 2wrg). The final images of the HA structures were generated using Pymol (Delano Scientific) [35].

## Results

### Characterization of 2009 pandemic H1N1 isolates in mice

**Figure 1:**
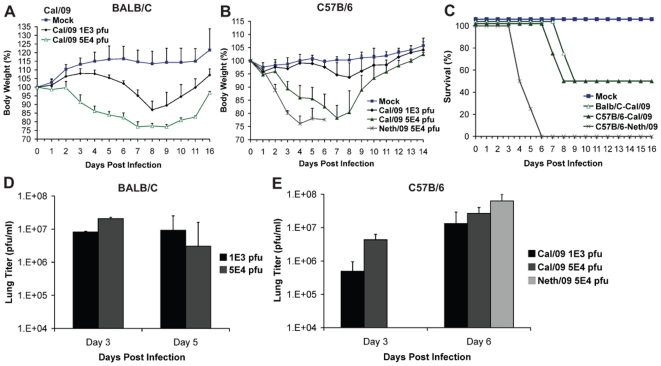
Differences in pathogenicity of 2009 pandemic H1N1 virus isolates in mice. (A) Body weight of BALB/c mice infected with the Cal/09 isolate. Six-week old BALB/c mice were infected with indicated dose of virus and body weights were monitored everyday as measure of virus induced disease. (B) Neth/09 isolate is more pathogenic than Cal/09 isolate in mice. C57B/6 mice were infected with either Cal/09 or Neth/602 isolate of 2009 pandemic H1N1 virus at indicated doses. (C) Comparison of survival rates of BALB/c and C57B/6 mice infected with Cal/04 and Neth/602 viruses. (D) Viral titers in the lungs of BALB/c mice infected with either 1×10^3^ or 5×10^4^ pfu of the Cal/09 isolate. (E) Viral titers in the lungs of C57B/6 mice infected with either 1×10^3^ or 5×10^4^ pfu of Cal/09 or 5×10^4^ pfu of Neth/09. ND indicates that data was not determined. The body weights were measured everyday and are represented as a percentage of body weight prior to infection (n = 4 or 5 mice/group). Mice showing more than 25% of body weight loss were considered to have reached the experimental end point and were humanely euthanized. The lungs of 2–3 infected mice were excised and viral loads in the homogenates were measured by performing plaque assay in MDCK cells.

To evaluate the virulence and infection kinetics of novel pandemic H1N1 viruses in mice, six-week old BALB/c and C57B/6 mice were infected with two virus isolates of 2009 H1N1 and monitored for signs of body weight loss and survival. Inoculation of BALB/c mice with Cal/09 isolate produced a marked decrease in body weight even at 10^3^ pfu (∼13%, Fig. 1A), a phenomenon not commonly observed with non-mouse adapted human H1N1 viruses [36]. When infected with 5×10^4^ pfu 50% of the mice reached the experimental end point of >25% weight loss by day 9 (Fig. 1C). The remaining mice showed substantial weight loss by day 8 p.i., with an average weight of 77.5% (Fig. 1A). Viral lung titers in these mice at both day 2 and 5 were notably high with no significant difference at both time points (Fig. 1D). Similar results were observed in C57B/6 mice after inoculation with Cal/09 virus. At doses of 10^3^ and 5×10^4^ pfu mice showed body weight losses comparable to those of BALB/c mice (Fig. 1B). The viral titers in the lungs of C57B/6 mice were almost ten-fold higher on day 6 p.i. than on day 3 p.i. (Fig. 1D), indicating active replication of virus. As with BALB/c mice, infection with 5×10^4^ pfu resulted in 50% of mice reaching the experimental end point by day 8 p.i. (Fig. 1C), and an average weight loss of 21.8% on day 7 p.i. (Fig. 1B).

To evaluate whether similar virulence is observed with other isolates of 2009 pandemic H1N1, we infected mice with the Neth/09 isolate. Interestingly, Neth/09 showed a considerably higher virulence in C57B/6 mice. At a dose of 5×10^4^ pfu/ml, the mice exhibited rapid and substantial weight loss by day 4 (Fig. 1B). All mice succumbed to infection or reached experimental end point by day 6 p.i. (Fig. 1C), and had higher lung viral titers than those observed with the Cal/09 isolate at this time point (Fig. 1E).

**Figure 2:**
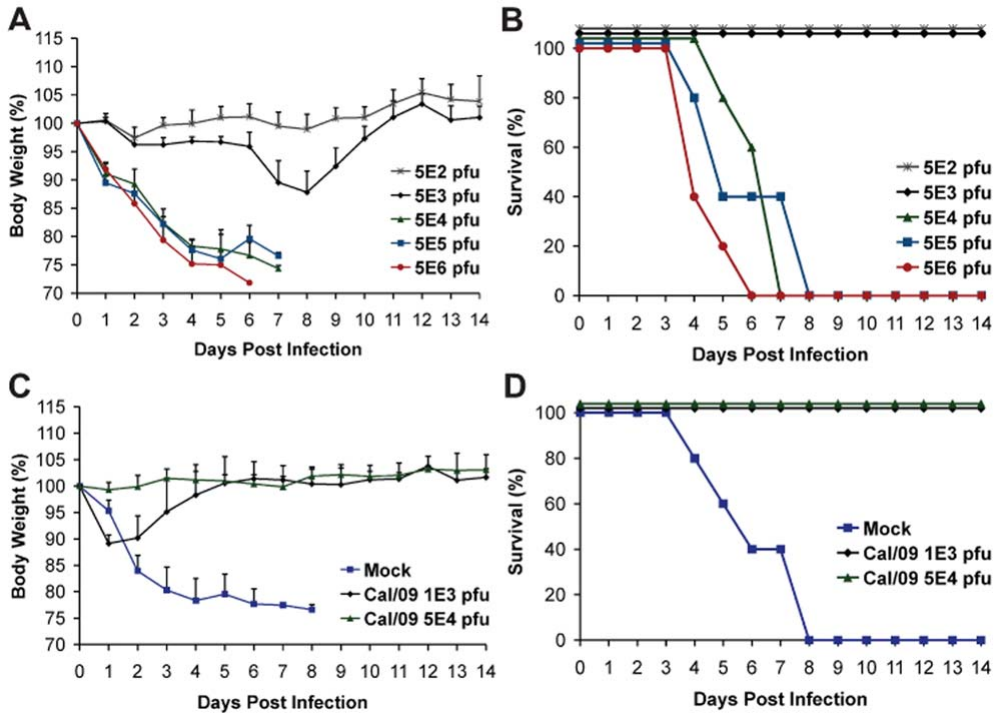
Prior infection with Cal/09 virus cross-protects mice against the lethal challenge with Neth/09 isolate. (A, B) Determination of LD_50_ for the Neth/09 isolate in C57B/6 mice (n = 5/group). Mice were infected with the indicated doses of virus and their body weight (A) and survival (B) were monitored for 14 days. (C, D) Re-challenge of Cal/09 infected mice with Neth/09 (n = 4/group). Six weeks old C57B/6 mice were inoculated with PBS (Mock) or Cal/09 isolate (10^3^ or 5×10^4^ pfu). After 21 days the mice were challenged with 1×10^6^ pfu of Neth/09 isolate. The body weight (C) and survival (D) were monitored for 14 days as in Fig. 1.

To further evaluate the pathogenesis of Neth/09, we next determined the LD_50_ of Neth/09 isolate in mice. Nine-week old female C57B/6 mice were infected with different doses of virus (5×10^2^, 5×10^3^, 5×10^4^, 5×10^5^ or 5×10^6^ pfu) and monitored for weight loss and survival over a period of 14 days p.i. (Fig. 2A and B). All mice inoculated with 5×10^4^ pfu or higher succumbed to infection or reached the experimental end point between days 4–8 p.i. (Fig. 2A). Mice inoculated with 5×10^3^ pfu showed a 12.2% decrease in body weight, and mice infected with 5×10^2^ pfu showed no significant drop in weight (Fig. 2B). The LD_50_ for Neth/09 was determined to be 1.58×10^4^ pfu for 9-week-old C57B/6 mice (Reed and Muench method, [33]). Overall, these data indicates that the Neth/09 isolate is more pathogenic in mice than Cal/09 and thus, in the challenge experiments described herein, the Neth/09 isolate and C57B/6 mice were used as a lethal model for 2009 pandemic H1N1.

### Antigenic cross-reactivity of 2009 pandemic H1N1 viruses

Next, to evaluate the level of antigenic resemblance between 2009 H1N1 viruses and other H1N1 viruses, we performed hemaglutination inhibiton (HI) assays and challenge experiments. Analysis of sera from mice infected with Cal/09 revealed similar levels of HI activity against the Cal/09 and Neth/09 isolates, suggesting that they are antigenically very similar, despite significant differences in pathogenicity in mice (data not shown).

As a proof of principle and to establish if prior infection with Cal/09 is sufficient to confer protection against a lethal challenge with an antigenically similar Neth/09 isolate, C57B/6 mice were first infected with Cal/09 and allowed to seroconvert for 21 days, followed by lethal challenge with 10^6^ pfu of Neth/09 (>50 LD_50_). As expected, all mice previously infected with Cal/09 survived (Fig. 2D). Mice previously inoculated with 10^3^ pfu of Cal/09 showed a decrease of 10% body weight. However, all the mice regained the weight by day 6 (Fig. 2C). No significant weight change was observed in the 5×10^4^ pfu group. In contrast, mice previously mock infected succumbed to Neth/09 challenge by day 8 (Fig. 2D). Together, this demonstrates that Cal/09 and Neth/09 are antigenically very similar and there is cross-protection between different 2009 pandemic H1N1 isolates.

### Vaccination with 1918-like or classical swine H1N1 viruses confers protection against Neth/09 lethal challenge

**Figure 3:**
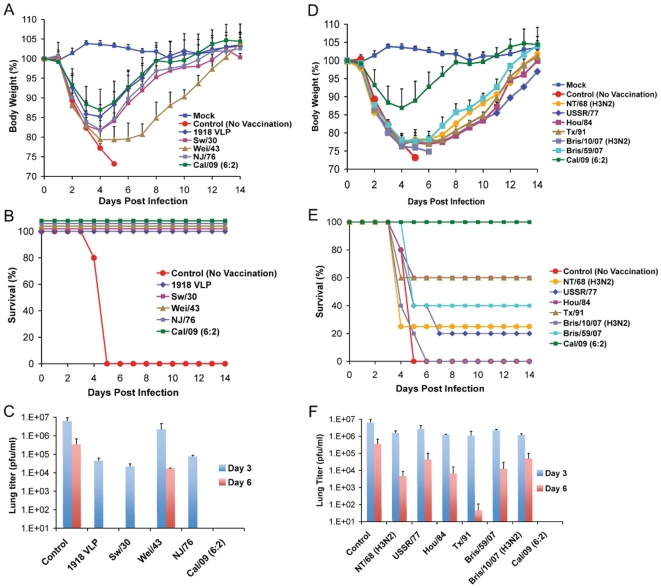
Vaccination of mice with 1918, 1943 and classical swine H1N1 virus-based inactive vaccines protects against Neth/09 lethal infection. Five weeks old C57B/6 were immunized with 15 µg of the indicated inactivated viruses or with 1918 VLP vaccine followed by a boost (15 µg) after two weeks. Four weeks after the first immunization, vaccinated mice were challenged with Neth/09 isolate at a dose of 50 LD_50_ (7.9×10^5^ pfu). Mice were monitored for body weight loss and survival for 14 days as in Fig. 1. (A) Body weight of classical swine H1N1 (Sw/30, NJ/76), 1918 VLP and human H1N1 Wei/43 vaccinated mice after challenge with Neth/09 (n = 4 or 5 mice/group) (D) Body weight of mice vaccinated with contemporary (from 1977–2007) H1N1 or H3N2 inactivated virus after challenge with Neth/09 (n = 4 or 5 mice/group). (B, E) Survival of mice vaccinated with H1N1 and H3N2 viruses post-challenge. (C, F) Viral titers in H1N1 and H3N2 vaccinated mice on days 3 and 6 p.c. Each data point represents the average viral titers from 2 or 3 mice. A single group of control (no vaccine) mice were used and are included in all the panels for direct comparison. The Cal/09 vaccinated group is also included on (A) and (B) for comparison.

Recent serological studies have shown that the human population under the age of 30 has little or no level of neutralizing antibodies against the 2009 pandemic H1N1 virus [22],[27],[28],[37]. However, sera from adults older than 35 years of age showed varied levels of neutralizing activity to 2009 pandemic H1N1, with people born before 1940's showing highest degree of neutralizing activity [27]. To investigate if antibodies against older H1N1 viruses can cross-react with and therefore offer protection against the 2009 pandemic H1N1 virus, C57B/6 mice were immunized with 1918 VLP or different inactivated H1N1 virus vaccines, spanning from 1918–2009. Prior to lethal challenge, all mice were tested for seroconversion against the homologous virus and were found to have HI antibody titers equivalent to ≥40 (Table 1). To test if vaccination with any of the other H1N1 viruses offered protection against novel 2009 pandemic H1N1, immunized mice were challenged with a lethal dose of Neth/09 (50 LD_50_), and the vaccine efficacy was evaluated by assessment of weight loss and survival over a 14 day period p.c., and also by measuring the virus titer in the lower respiratory track (Fig. 3). In the no vaccination group (control), mice succumbed to infection and reached the experimental endpoint by day 5 (Fig. 3B). This group of mice showed the highest viral titers in the lungs at days 3 and 6 p.c. (Fig. 3C). As anticipated immunization with Cal/09 6:2 offered full protection from death to mice (Fig. 3B). Modest levels of disease were observed in this group as evidenced by a decrease in body weight (Fig. 3A). However, we did not detect infectious virus in the lungs of these mice on days 3 or 6 p.c. (Fig. 3C; limit ≥10 pfu). Interestingly, inactivated classical swine H1N1 viruses (Sw/30 or NJ/76), 1918 VLPs and an inactivated human H1N1 virus isolated in 1943 (Wei/43) offered 100% protection against death from 2009 pandemic H1N1 challenge. The 1918 VLP-vaccinated mice all survived and showed weight loss comparable to that of the Cal/09 6:2 vaccinated animals (Fig. 3B and 3A). We detected 100-fold lower levels of virus in the lungs of challenged mice on day 3 p.c. and no virus on day 6 p.c. Similarly, in mice immunized with Sw/30 or NJ/76, Neth/09 virus was only detected in the lungs on day 3 p.c., indicating that these two vaccines had an efficacy in reducing viral replication in lungs comparable to that of 1918 VLPs (Fig. 3C). However, a slightly higher weight loss (∼18.2%) was observed in these groups (Fig. 3A). Although vaccination with Wei/43 resulted in 100% survival, these mice showed a much greater and sustained weight loss than the 1918 VLP, SW/30 and NJ/76 vaccinated mice, and the viral titers in the lungs were high on both day 3 and day 6 p.c., suggesting only modest levels of inhibition of Neth/09 virus replication (Fig. 3A, B and C). In contrast, immunization with human H1N1 viruses that circulated from 1977–2007 showed only partial protection against lethal Neth/09 challenge (Fig. 3E). This was evidenced by extensive weight loss observed in these mice (Fig. 3D). In mice vaccinated with USSR/77, Hou/84, Tx/91 or Bris/59/07 only 20–60% survival was observed (Fig. 3D and E). The viral titers on day 3 p.c. were similar to controls. Nevertheless, on day 6, viral titers were lower, particularly in the Tx/91 vaccinated mice that had around 1000-fold lower viral titers as compared to day 3 p.c. (Fig. 3F). In the groups vaccinated with the NT/68 and Bris/10/07 strains, viruses belonging to the H3N2 subtype, lethal challenge resulted in either a 25% survival or all mice succumbing to infection, respectively. In agreement with the increased morbidity seen in Bris/10/07 vaccinated mice, weight loss and viral titers were also high. The reasons for a lower viral titer in the NT/68 vaccination group on day 6 p.c. are unclear (Fig. 3F). Nevertheless, these results indicate that inactivated vaccines based on classical swine H1N1 viruses and on human 1918 and 1943 H1N1 viruses protect against death in mice infected with the 2009 pandemic H1N1 virus and suggest that they likely share significant antigenic similarity.

**Table 1:** HI activity of mice sera against homologous virus post-vaccination.

Virus	HI titer^a^^,^ b (Pre-challenge)
1918 VLP	40–320
Sw/30	40–160
Wei/33	640–1280
NT/68	40–640
NJ/76	80–320
USSR/77	640
Hou/84	80–640
Tx/91	160–640
Bris/59/07	80–640
Bris/10/07	320–640
Cal/09	40–2560

a Range of HI antibody titer of 10 vaccinated mice per group.

b HI titers represent the highest dilution that displayed hemagglutination inhibitory activity.

Next, to test whether the protection observed in 1918–1943 and NJ/76 vaccinated correlates with cross-reactive neutralizing antibodies in the sera, we tested the pre-challenge sera from vaccinated mice for HI activity against different H1N1 viruses (Table 2). All the pre-challenge sera showed HI titer between 160–2560 against the homologous virus. As expected, sera from 1918 VLP, SW/30 and NJ/76 vaccinated animals showed HI activity against both 2009 H1N1 pandemic viruses (HI titer ≥40), suggesting that protection is due to cross-reactive antibodies. However, despite complete protection against death from Neth/09 lethal challenge, the sera from Wei/43 showed no HI activity against 2009 H1N1 viruses. This might indicate that the HI assay is less sensitive than the challenge assay *in vivo* to evaluate the presence of protective Abs. Taken together, our results demonstrate that 1918, SW/30 and NJ/76 share antigenically similar epitope(s) that might be responsible for the cross-protection.

**Table 2:** HI activity of polyclonal sera from mice vaccinated against different H1N1 isolates.

	HI titer of mouse sera^a^^,^ b											
Virus	1918 VLP	Sw/30	Wei/43	NT/68	NJ/76	USSR/77	Hou/84	Tx/91	Bris/59/07	Cal/09	Neth/09^c^	Control^d^
1918 VLP	**160**	**40**	<10	<10	**20**	<10	<10	<10	<10	**320**	**80**	<10
Sw/30	**20**	**160**	<10	<10	**80**	**80**	<10	<10	<10	**10**	<10	<10
Wei/33	<10	<10	**1280**	<10	<10	<10	**20**	<10	<10	<10	<10	<10
NT/68^e^	<10	<10	<10	**640**	<10	<10	<10	<10	<10	<10	<10	<10
NJ/76	**10**	**80**	<10	<10	**320**	<10	<10	<10	<10	**80**	<10	<10
USSR/77	<10	<10	<10	<10	<10	**640**	<10	<10	<10	<10	<10	<10
Hou/84	<10	<10	<10	<10	<10	**40**	**320**	<10	<10	<10	<10	<10
Tx/91	<10	<10	<10	<10	<10	<10	<10	**640**	<10	<10	<10	<10
Bris/59/07	<10	<10	<10	<10	<10	<10	<10	<10	**640**	<10	<10	<10
Cal/09	**160**	**80**	<10	<10	**40**	<10	<10	<10	<10	**2560**	**640**	<10
Neth/09	**160**	**80**	<10	<10	**40**	<10	<10	<10	<10	**640**	**1280**	<10

a HI titers represent the highest dilution that displayed hemagglutination inhibitory activity.

b Positive titers are bolded for clarity.

c HI activity of sera from mice infected with Neth/09 is included for comparison.

d Control- negative sera.

e H3N2 strain representative used in the vaccination studies.

### Passive immunity with 1918 mAb protects against Neth/09 lethal challenge

**Figure 4:**
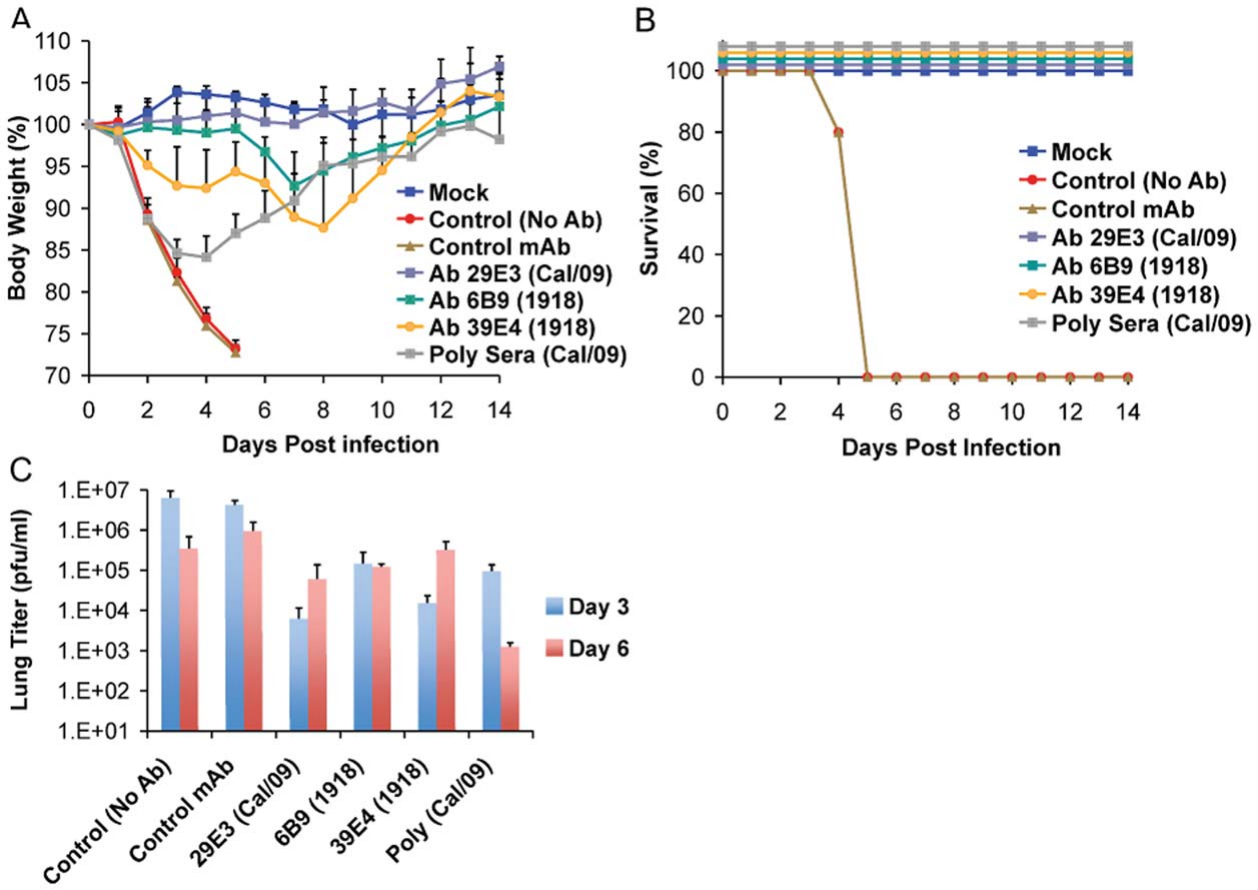
Passive immunization of mice with 1918 and 2009 H1N1 HA specific cross-reactive antibodies protects against Neth/09. Nine week old, C57B/6 mice were passively immunized with a total 150 µg of indicated monoclonal antibody (intraperitoneal route) 24 hr prior to viral challenge. After antibody administration, the mice were challenged with Neth/09 isolate at a dose 50 LD_50_. (A) Body weight of passively immunized mice challenged with Neth/09 (n = 5/group). (B) Survival of passively immunized mice post-challenge with Neth/09. (C) Viral Titers in the lungs at days 3 and 6 p.c. Viral load in the lungs homogenates were quantified as in Fig. 1. Control (No Ab) mice are the same as controls in Fig. 3 and are included for direct comparison.

To further examine the common possible epitopes shared between 1918 and 2009 H1N1 viruses, we examined cross-reactivity of 1918 HA-specific monoclonal antibodies with Cal/09 by HI assay. Two 1918 HA mAb's, 6B9 and 39E4, showed HI activity against Cal/09. Also, one of the Cal/09 HA specific antibodies (29E3) showed HI activity against Cal/09 and against 1918 VLPs (Table 3). To evaluate if these mAbs could confer protection (*in vivo*) against lethal Neth/09 challenge, mice were given 150 µg of mAb as a prophylactic treatment 24h prior to challenge (50 LD_50_). To assess the level of HI titers in the blood prior to challenge, the HI activity in passively immunized mice sera was measured (Table 4). Sera from mice immunized with an Ab raised against an unrelated viral protein (Nipah virus W) did not show HI activity. Mice immunized with HA-specific monoclonal antibodies showed considerable levels of HI titers (HI = 40–320). Mice immunized with polyclonal sera showed a lower HI titer (HI = 20–80; Table 4). As expected, administration of the control antibody resulted in no protection from lethal challenge and all mice had high virus titers in the lungs equivalent to those seen in untreated mice (Fig. 4). Polyclonal sera from mice previously infected with Cal/09 virus were used as a positive control prophylactic treatment to establish the baseline of protective antibodies *in vivo* (Poly sera). Mice in this group were completely protected from lethal challenge and showed a decrease in lung virus titers on day 3 and 6 compared to untreated controls. Nonetheless, these mice showed a significant weight loss during the first 4 days (Fig. 4A, B and C). Treatment with 150 µg of 1918 specific anti HA monoclonal antibodies (6B9 and 39E4) also conferred full protection from challenge and showed reduced weight loss as compared to the polyclonal sera treatment group. The Cal/09 specific monoclonal antibody (29E3) treatment resulted in 100% survival and no weight loss, indicative of little or no morbidity in these mice. Assessment of infectious virus in the lower respiratory track of mice treated with all monoclonal antibodies revealed decreased viral titers as compared to controls especially on day 3 p.c. (Fig. 4C). Taken together, these data suggest that cross-reactive monoclonal antibodies protect against lethal Neth/09 challenge by blocking a conserved antigenic site between 1918 and 2009 pandemic H1N1 viruses.

**Table 3:** HI activity of anti-HA monoclonal antibodies used for passive immunity treatment.

Antigen specificity	Antibody	Cal/09 6:2	Bris/59/07	1918 VLPs
**1918 HA** a	6B9	0.15	>5	0.15
	39E4	0.15	>5	0.15
**Cal/09 HA** a	29E3	0.04	>5	0.08
**Nipah W** a	Control	>5	>5	n/d^b^

a Specific HI activity of mAbs was calculated as the lowest concentration (µg/ml) of mAb that displayed hemagglutination inhibitory activity.

b n/d: not determined.

**Table 4:** *In vivo* HI titers in passively immunized mice pre-challenge.

Antigen Specificity	Antibody	HI Titers^a^
**1918 HA**	6B9	n/d^b^
	39E4	40–320
**Cal/09 HA**	29E3	160–320
**Nipah W**	Control	<10
**Cal/09 virus**	Sera	20–80
**N/A** b	Sera (Negative)	<10

a HI titers represent the highest dilution that displayed hemagglutination inhibitory activity.

b N/A: Not applicable, n/d: not determined.

### Mapping of cross-protective epitopes

**Figure 5:**
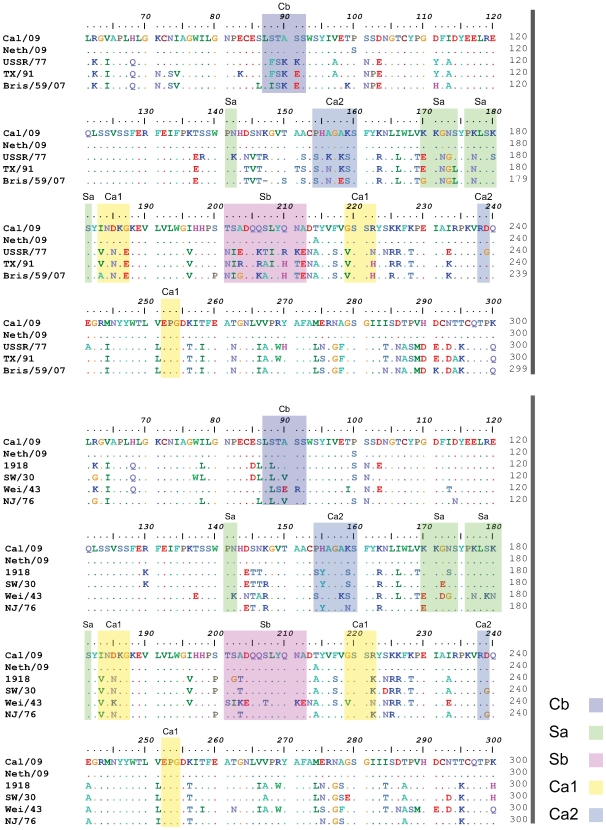
Comparison of the conservation of antigenic sites in HA of H1N1 viruses. Alignment of HA sequences from USSR/77, Tx/91, Bris/59/07 and 2009 human H1N1 viruses (top panel). Alignment of HA sequences from 1918, Sw/30, Wei/43, NJ/76 and 2009 H1N1 viruses (bottom panel). The antigenic sites are indicated in colored shaded boxes and were previously described by Brownlee and Fodor [42], using H3 numbering, as Cb (78–83), Sa (128–129, 156–160, 162–167), Sb (187–198), Ca1 (169–173, 206–208, 238–240) and Ca2 (140–145, 224–226).

**Figure 6:**
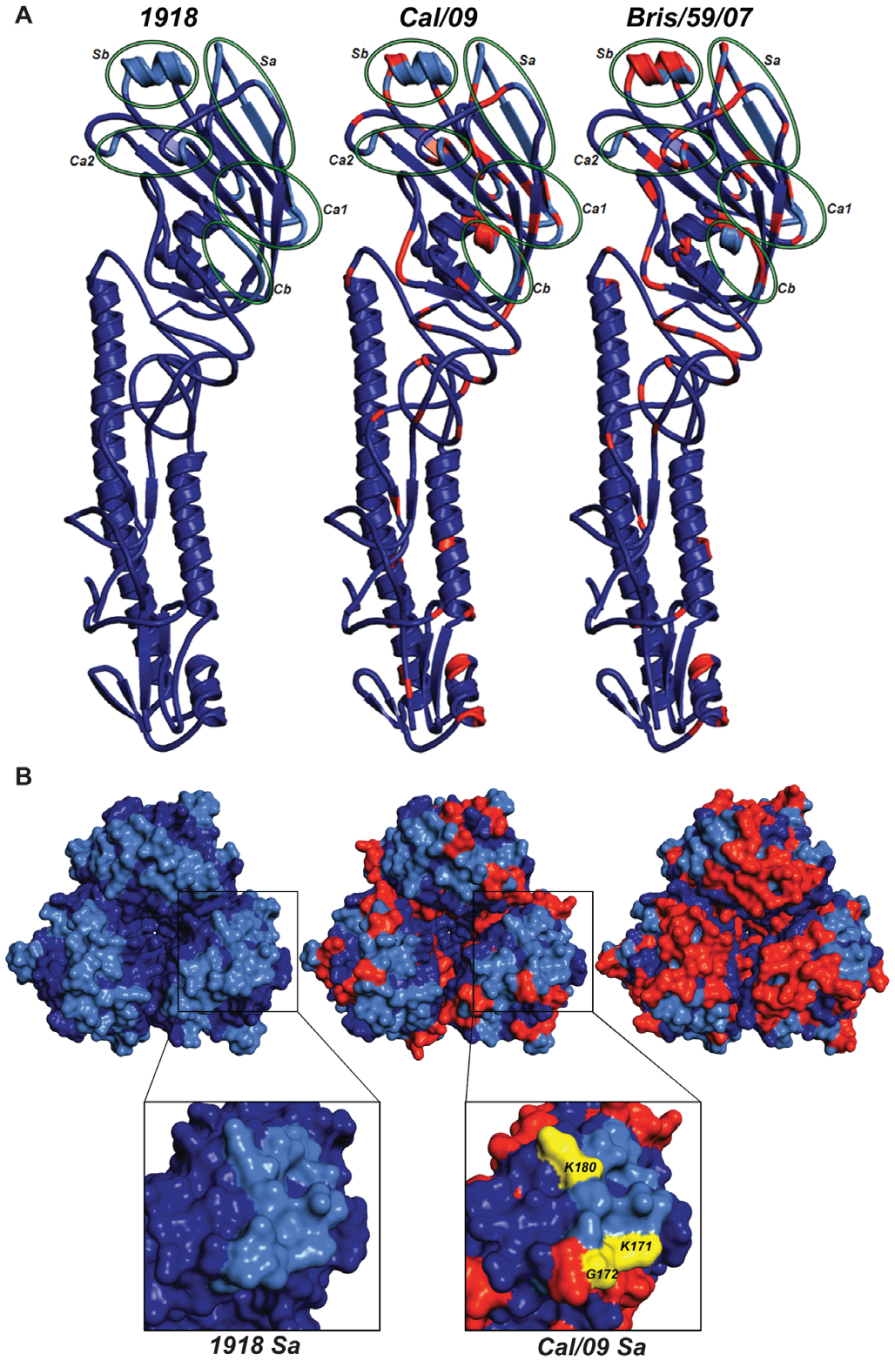
Comparison of antigenic differences in the HA structures of 1918, Cal/09 and Bris/59/07 viruses. (A) Ribbon representation of an HA monomer (H1 and H2) of the three viruses. Individual antigenic sites are highlighted by green ellipses in the HA monomers. (B) Top panel: Zenithal surface top view of HA trimeric complexes depicting antigenic sites and their differences among 1918, Cal/09 and Bris/59/07 viruses. Blue color corresponds to conserved amino acids and red color represents amino acids that differ from 1918 HA. The antigenic sites are colored in light blue. Bottom panel: Close-up view of antigenic site Sa in monomeric 1918 and Cal/04 HAs. Amino acids K171, G172 and K180 (K157, G158 and K166 by H3 numbering) corresponding to mAb escape mutations are shown in yellow.

To identify the shared cross-protective epitope(s) in HA, we generated mAb escape mutants of Cal/09 6:2 virus. Escape mutants generated by pre-incubation of Cal/09 virus (6:2) with excess of each of the cross-reactive mAbs (6B9, 39E4 and 29E3) resulted in a loss of HI activity against the same mAb. Of interest, all the escape mutant viruses generated with either 1918- or 2009 HA-specific mAbs carried mutations in the antigenic site Sa (Table 5; Fig. 5). The escape mutants that arose in the presence of the 1918 specific mAb 6B9 and 39E4 contained mutations G172E (G158E by H3 numbering) and K171E (K157E by H3 numbering), respectively. The escape mutants generated by selection with the 2009 H1N1 specific mAb (29E3) carried either a K171E or K171Q or K180N mutation (residues K171 and K180 correspond to K157 and K166 in H3 numbering). These results indicate that all the mAbs bind to the conserved antigenic site Sa (Fig. 5 and 6).

**Table 5:** Cross-reactive mAb escape mutants of Cal/09.

Antigen Specificity	Antibody	Mutation^a^
**1918 HA**	6B9	G172E (G158E)
	39E4	K171E (K157E)
**Cal/09 HA**	29E3	K171E (K157E)
		K171Q (K157Q)
		K180N (K166N)

a H3 numbering are shown in parenthesis.

## Discussion

Seasonal influenza viruses predominantly cause severe disease in very young children and in the older population. However, infections with the 2009 pandemic H1N1 are considerably lower in people 65 years or older, likely due to immunity from prior exposure and/or vaccination to an antigenically similar influenza virus [24],[25],[26]. Using a panel of 11 different influenza viruses from 1918–2009, we tested the ability of inactivated vaccines based on these viruses to protect against a mouse-lethal 2009 pandemic H1N1. Here, we show that vaccination of mice with human 1918 influenza virus VLPs, and inactivated human Wei/43 and classical swine H1N1 viruses completely protects from death by lethal challenge with the 2009 pandemic H1N1. Also, analysis of pre-challenge sera from mice vaccinated with these viruses, except Wei/43, show cross-reactivity against the 2009 H1N1 virus (HI titer ≥40). In contrast, vaccination with more contemporary H1N1 viruses offered only partial protection. In addition, passive immunization with 1918 HA-specific monoclonal antibodies protects against the 2009 H1N1 virus, suggesting antigenic similarity between these viruses. Cross-protective epitope mapping shows that 1918 HA-specific mAbs protect by binding to the conserved antigenic site Sa in 2009 H1N1 virus. Based on our HI data (Table 2) and since inactivated vaccines are known to induce protective humoral and minimal cellular immunity, this protection was likely mediated by antibodies, as also evidenced by the lack of complete protection by H3N2-based vaccines. Our results indicate that prior exposure to human influenza A viruses from 1918–1943 or vaccination with classical swine H1N1 virus (NJ/76) offers significant levels of cross-protection against the novel 2009 H1N1 pandemic virus and thus provides a mechanistic explanation for the lower incidence of disease and/or infection seen in people aged 65 or older.

Usually human influenza viruses do not show substantial replication in mice without prior adaptation to this host [36]. Only a few viruses including 1918 and highly pathogenic H5N1 viruses have been shown to replicate efficiently and cause severe pathogenicity [38]. The current 2009 pandemic H1N1 shows active replication and pathogenesis in mice. However, there appear to be differences in the pathogenicity and transmission of 2009 H1N1 isolates in different animal models [39],[40],[41]. In this study we have established a lethal mouse model for the 2009 pandemic H1N1 virus and demonstrate differences between the Cal/09 and Neth/09 viruses in terms of mouse virulence. Mice infected with Neth/09 showed an early and dramatic loss in body weight and succumbed to infection or reached the experimental end point by day 6 (Fig. 1B and C; Fig. 2A and B). Cal/09 and Neth/09 viruses were both isolated from humans showing mild upper respiratory symptoms and their genomes only differ at eight amino acid positions [39],[40]. The reasons for the differences in mouse virulence between these two isolates remains unclear. Despite these differences, antigenicity based on HI cross-reactivity between the two virus isolates is similar. This is in agreement with recent reports from the World Health Organization stating that all currently circulating 2009 H1N1 pandemic isolates are antigenically similar (www.who.int).

Although at this moment we can not exclude the possibility that the NA protein contributed at least in part to the protection seen with the inactivated vaccines, our results from the passive immunization and vaccination studies suggest that human H1N1 viruses from or prior to 1943, including the 1918 virus, share common protective epitope(s) in the HA protein. The antigenic sites of influenza virus HA's has been extensively characterized by monoclonal antibody epitope mapping studies [32],[42]. Interestingly, sequence alignment of the HAs from 1918, Sw/30, NJ/76 and 2009 H1N1 viruses showed a high degree of similarity in the known antigenic sites (Fig. 5, bottom). To further understand the antigenic relatedness between 1918 and 2009 H1N1 viruses, we compared the structure of the 1918 HA with the predicted HA structures of Cal/09 and Bris/59/07 (Swiss-Model; Fig. 6A). It is apparent that the globular head regions of HA's, that harbor the known antigenic sites, share close similarities between the Cal/09 and 1918 viruses (Fig. 6A and B). However, the antigenic sites in Bris/59/07 HA contain several amino acid changes as compared to the 1918 HA (evidenced from the higher number of red colored residues, Fig. 6B, top panel). This is supported by analysis of the pre-challenge sera from mice vaccinated with 1918 VLP that show high cross-reactivity against 2009 H1N1 virus (Table 2). Although the Wei/43 vaccine conferred complete protection from death, Wei/43 HA shows less sequence similarity to the 2009 H1N1 HA in the antigenic sites as compared to 1918 and the sera from Wei/43 vaccinated animals showed no cross-reactivity with 2009 H1N1 viruses by HI assay (Fig. 5; Table 2). In agreement with our findings, Wei/43 vaccinated animals showed more morbidity and higher viral titers after Neth/09 challenge than Cal/09 and 1918 vaccinated mice. Thus, Wei/43 appears to have a slightly decreased protective effect, suggesting that circulation of H1N1 viruses in humans for approximately 2 decades (after the 1918 pandemic) resulted in sufficient drift leading to considerable loss of antigenic cross-reactivity. One would therefore predict that H1N1 viruses that have circulated in the human population beyond the 1950's would offer little or no protection against the 2009 pandemic H1N1. Although, there is a correlation between sequence identity in the antigenic sites and protection, it is possible that some of the changes in the antigenic sites may not contribute to the overall alterations in antigenicity. In contrast to 1918 and classical swine H1N1 viruses, alignment of 2009 H1N1 with H1N1 HAs from viruses isolated in humans after 1977 showed significant variations in the antigenic sites especially in sites Sa and Sb (Fig. 6A, top). This is in agreement with our results showing that contemporary H1N1 virus vaccines only offered partial protection in mice against Neth/09 challenge and that surviving mice showed greater morbidity as evidenced by a more dramatic and sustained weight loss (Fig. 3D). Of interest, two of the more recent viruses, Tx91 and Hou/84, showed nearly 60% protection from death in mice to Neth/09 challenge. However, the reasons for this better partial protection offered by these two virus vaccines are unclear. Nevertheless, similar partial protection from lethal challenge has been previously described for antigenically different H1N1 viruses [43]. The mechanism for this partial protection is unclear and has been speculated to be largely due to anti-HA immunity [43]. Also, we cannot rule out completely the possible roles of T-cell mediated immunity or anti-NA-specific antibodies [44],[45],[46],[47],[48]. This warrants further investigation.

To characterize the antigenic similarity, we generated mAbs against the HA of 1918 and 2009 H1N1 viruses. Interestingly, two 1918 HA-specific mAbs, 6B9 and 39E4, and one Cal/09 HA specific mAb, 29E3, showed HI activity against both 1918 VLP and Cal/09 virus (Table 4). Passive immunization of mice with both 1918 HA-monoclonal antibodies afforded complete protection against lethal Neth/09 challenge and a 100–1000 fold reduction in viral titers in the lower respiratory track of mice. However, we did notice a loss in body weight starting on day 4–6 and an increase in lung titers on day 6, likely due to waning of neutralizing antibody levels in the sera. In the case of polyclonal sera treatment, the HI titers in the blood prior to challenge were 4-fold lower than monoclonal antibody treatment. In agreement with the lower HI titer, we observed an initial decrease in body weight up to 4 days following Neth/09 challenge. All these mice started recovering weight by day 5. Despite lower HI titers in the sera prior to challenge, the polyclonal sera treated mice had lung titers 1000 fold lower than control mice. This could be due to the action of NA specific antibodies which have been demonstrated to play a protective role [46], and to other HA specific antibodies without HI activity. The ability of 1918 HA specific antibodies to neutralize the 2009 pandemic H1N1 further confirms the close antigenic relationship of these viruses. Indeed, by generating antibody escape mutant viruses of Cal/09, we mapped antigenic site Sa as the cross-reactive epitope (Table 5 and Fig. 6B). Overall, this highlights the apparent slower pace of antigenic drift of the classical swine H1N1 viruses since their introduction into the pig population over 90 years ago, most likely due to the absence of pre-existing immunity in the majority of this population.

In 1976, an H1N1 virus closely related to classical swine H1N1 isolates from the 1930's caused an influenza outbreak at an American military facility, Fort Dix, in the state of New Jersey. After the 1976 swine H1N1 outbreak, nearly 40 million people in the United States were immunized with an NJ/76 vaccine. Our study in mice, in agreement with previous serology done with ferret sera [49], shows that immunization with NJ/76 vaccine protected from 2009 pandemic H1N1 lethal challenge, and therefore it is likely that people carrying antibodies against the NJ/76 will be protected against the 2009 H1N1. Additionally, sera from adults immunized with NJ/76 vaccine cross-neutralized the 2009 H1N1 [27].

Two recent human serology studies showed a high prevalence of neutralizing antibodies against 2009 pandemic H1N1 in people born before 1930 [28],[39]. Our data reveals that immunization with human H1N1 viruses that circulated before 1945 (e.g. specific antibodies against 1918 and Wei/43) is sufficient for immune protection from the 2009 pandemic H1N1. These findings provide a rationale for the epidemiological data arising from different parts of the world that have indicated a consistently higher rate of infection with this novel virus in the younger population (<35 of age), due to the lack of previous exposure to any of the antigenically-related viruses described above. As such, these results greatly emphasize that vaccination efforts and resources should also be directed at this susceptible population.

Our data are consistent with a picture in which domestic pigs have served as a reservoir for an “antigenically frozen” H1 hemagglutinin derived from the 1918 influenza virus. A significant drift in humans of that same 1918 H1N1 virus has resulted in a lack of significant pre-existing immunity against the 2009 pandemic H1N1 virus in humans born after the 1940–50s. Of concern, swine influenza viruses containing HA genes derived from more modern human H3 and H1 viruses have also established lineages in North American pigs during 1997–1998 and 2003–2005, respectively [13]. Also, human H3 viruses have established stable lineages in European swine populations after the 1968 “Hong Kong” pandemic, and are still circulating in the form of human-avian H3N2 reassortant viruses [50]. It is possible that these new swine viruses would also remain “antigenically frozen”, leading to potential human pandemic H3 and H1 viruses in the future [51],[52]. As such, surveillance and containment of swine influenza viruses is desirable for the prevention of future pandemic episodes.
